# Utility of Preoperative Inflammatory Markers to Distinguish Epithelial Ovarian Cancer from Benign Ovarian Masses

**DOI:** 10.7150/jca.51642

**Published:** 2021-03-05

**Authors:** Lian Li, Jing Tian, Liwen Zhang, Luyang Liu, Chao Sheng, Yubei Huang, Hong Zheng, Fengju Song, Kexin Chen

**Affiliations:** 1 Department of Epidemiology and Biostatistics, National Clinical Research Center for Cancer, Key Laboratory of Cancer Prevention and Therapy of Tianjin, Tianjin Medical University Cancer Institute and Hospital, Tianjin, China.; 2 Department of Urology, National Clinical Research Center for Cancer, Key Laboratory of Cancer Prevention and Therapy of Tianjin, Tianjin Medical University Cancer Institute and Hospital, Tianjin, China.

**Keywords:** Epithelial ovarian cancer, Inflammation biomarkers, Diagnosis, Benign ovarian masses, Cancer biomarkers.

## Abstract

**Background:** Inflammatory markers have been reported to be predictors for the presence of epithelial ovarian cancer (EOC), however, the cut-off value of each marker remains unclear and predictive capability of the markers in different histology types of EOC is still unknown.

**Methods:** A total of 207 patients with benign ovarian masses and 887 EOC patients who underwent surgical resection, and were pathologically diagnosed were included. We compared the difference of preoperative inflammatory markers between benign ovarian masses and EOC patients. Stratified analysis by histology subtype was further conducted. Logistic regression analyses and receiver operating characteristic (ROC) curves was used to evaluate the predictive capability of the markers.

**Results:** Neutrophil-to-lymphocyte ratio (NLR), platelet-to-lymphocyte ratio (PLR), and lymphocyte-to-monocyte ratio (LMR) were significantly associated with all stages and subtypes of EOC (P<0.001). The optimal cut-off points based on ROC curve analyses for NLR, PLR, and LMR were found to be 2.139 (AUC=0.749, *P*<0.001), 182.698 (AUC=0.730, *P*<0.001), and 3.619 (AUC = 0.709, *P*<0.001), respectively. In low CA125 level patients, high level of NLR and PLR increase the risk of endometrioid EOC, while low level of LMR were significantly associated with an increased risk of serous EOC.

**Conclusions:** In addition to CA125, NLR, PLR, and LMR could be used as predictors of EOC and preoperative inflammatory markers may be used as a potential biomarker for predicting different histotypes of EOC.

## Introduction

Epithelial ovarian cancer (EOC) is a major type of ovarian cancer and is the leading cause of death in patients with gynecologic malignancies 1. Among adnexal masses, nearly 75% are benign, 15% are EOC, and 10% are borderline or other types of malignant ovarian cancers 2, 3. Histological subtypes of EOC including serous, clear cell, endometrioid, and mucinous carcinoma et al. Benign ovarian masses have much better prognosis than EOC and the tumor pathology and differentiation between benign ovarian masses and EOC affect the subsequent treatment decision 4. However, it is difficult to distinguish benign and malignant prior to surgery when ovarian masses are found.

Besides patient's age, clinical symptoms, physical examination, the most widely used clinical tools to distinguish benign and malignant of adnexal masses are imaging modalities and tumor markers. Several algorithms such as RMI 5, 6, risk of ovarian malignancy algorithm (ROMA) 7, and the multivariate index assay (OVA1) 8 have been used to evaluate the malignancy potential of adnexal masses. Cancer antigen 125 (CA125) is the most widely used tumor marker in ovarian cancer 5, 9, However, the sensitivity and specificity of CA125 for the diagnosis of EOC is not ideal and CA125 alone should not be used to distinguish between benign and malignant adnexal mass 10. Therefore, it is necessary to identify additional biomarkers which combined with CA125 to improve the diagnosis accuracy of presence EOC.

Inflammation has been reported to play important role in carcinogenesis and cancer progression 11. Several inflammation related markers, including neutrophils to lymphocyte ratios (NLR), platelet to lymphocyte ratios (PLR), and lymphocyte to monocyte ratio (LMR) et al. have been shown to be a diagnosis or prognosis makers of cancers 12-16. These makers could be acquired from routine blood test, which known to be cost-effective, reproducible, less invasive, and universally accepted test currently. Previous studies have also reported that these inflammatory markers could distinguish benign ovarian masses and ovarian cancers 17-20. However, the cut-off value remains unclear and the accuracy of diagnosis in different histology types of EOC remains unknown 18, 20, 21. Therefore, we conducted a large sample size retrospective study to identify new biomarkers to assist CA-125 in distinguishing benign masses and EOC. In this study, we propose the optimal cutoff of NLR, PLR, and LMR to predict the presence of EOC, and evaluate the prediction performance in different histology types of EOC and provide evidence for clinical practice.

## Materials and methods

### Populations

Patients who underwent primary surgery in the department of Gynecological oncology at Tianjin Medical University Cancer Institute and Hospital between 2007 and 2017 were recruited. A total of 1707 patients were included according to following inclusion criteria: (1) underwent primary surgery and histologically confirmed for EOC or benign ovarian masses; (2) conducted blood test preoperative routine blood test (2 weeks before surgery), and CA125 information. Patients were excluded if they (1) underwent radiotherapy, chemotherapy, targeted drugs, or other anti-tumor therapy prior to surgery (280 patients); (2) had a history of autoimmune diseases or other malignancies (152 patients). (3) had borderline ovarian cancer (164 patients). (4) had no demographic information (17 patients). Finally, a total of 1094 patients (207 patients with benign ovarian masses and 887 EOC patients) were included for the analysis. Informed consent was obtained for use of the medical records for research purposes and this study was approved by the institutional review board (IRB) of Tianjin Medical University Cancer Institute and Hospital.

### Medical records data

Demographic data, clinical data, pathology report, and laboratory report were retrospectively obtained from medical records. Body mass index (BMI) refers to weight (kg) divided by the square of height (m). Tumor stage was divided into four stages (I, II, III, and IV) according to International Federation of Gynaecology and Obstetrics (FIGO) 2009 Criteria. Then we further classified into early stage (I and II) and advanced stage (III and IV) groups. Pathological diagnosis was divided into two groups: benign ovarian masses (serous cystadenoma, mucinous cystadenoma, mature teratoma, etc.) and epithelial ovarian cancer (serous ovarian cancer, mucinous ovarian cancer, endometrioid ovarian cancer, and other epithelial ovarian cancer). Tumor diameter was classified into two groups of <5cm and ≥5cm. Optimal cytoreduction was considered as the residual tumor size ≤1cm.

Blood cell counts were obtained from the routine blood test that was conducted within a week before operation. NLR refers to the ratio of the absolute neutrophils count to absolute lymphocyte count. PLR refers to the ratio of the absolute platelet count to the absolute lymphocyte count. LMR refers to the ratio of the absolute lymphocyte count to the absolute monocyte count.

### Statistical analysis

To compare the demographic characteristics and preoperative markers between the benign and malignant groups, Student's t-test was used to analysis the variables with normal distributions, and Mann-Whitney U-test was used to analysis the variables with non-normal distributions. Categorical variables were presented as n (%) and analyzed using χ^2^ test. Logistic regression analyses were used to evaluate the association between the biomarkers and malignancy risk. The receiver-operating characteristic (ROC) curve analysis was used to assess the discriminative role of markers and determine the appropriate cut-off values. Subgroup analysis was performed according to stage, histological subtypes and menopausal status. A two-tailed* P* value<0.05 was considered statistically significant. All statistical analysis was performed using SAS 9.4 and R version 3.4.3.

## Results

### Patient characteristics

This study included 1094 patients in total, 207 patients were benign ovarian masses and 887 of whom were EOC. The baseline characteristics of the patients were shown in Table S1 and Table S2. The significant difference was observed in age and menopause status between benign ovarian masses group and EOC groups (*P*<0.001). And there was no significant difference between the two groups in other demographic and life style factors including BMI, family history of cancer, smoking, and age of menarche et al. The histological subtypes of most EOC patients presented with serous (*n*=477, 53.78%) and endometrioid (*n*=194, 21.87%). Serous cystadenoma and mucinous cystadenoma accounted for 15.94% (n=33) and 5.31% (n=11) in terms of benign ovarian masses, respectively.

### Comparison of variables between benign ovarian masses and EOC groups

The median levels of CA125, HGB, WBC, NLR, PLR, and LMR were significantly different between benign masses and EOC groups (All *P*<0.001) (Table 1). In addition, the significant differences were also observed in these variables in the stratified analysis according to histological subtypes of EOC compared to benign ovarian masses (All *P*<0.001) (Table 2). Univariate and multivariate logistic regression model showed that the aforementioned markers were significantly associated with the presence of EOC (Table S3).

### ROC curve analyses

Receiver operating characteristic curves for EOC prediction were presented in Table 3. CA125 performed best for the discrimination of benign from EOC (AUC=0.906, 95%CI: 0.885-0.928), followed by NLR (AUC=0.749, 95%CI: 0.714-0.784), PLR (AUC=0.730, 95%CI: 0.696-0.764), and LMR (AUC=0.709, 95%CI: 0.672-0.745). Moreover, PLR, NLR, and LMR still have good performance in distinguishing benign and malignant ovarian masses after adjusting for age (Table S4) and in stratified analysis by stage, histological subtypes, and menopausal status (Table S5- Table S10, Figure S1- Figure S3).

**Figure 1:**
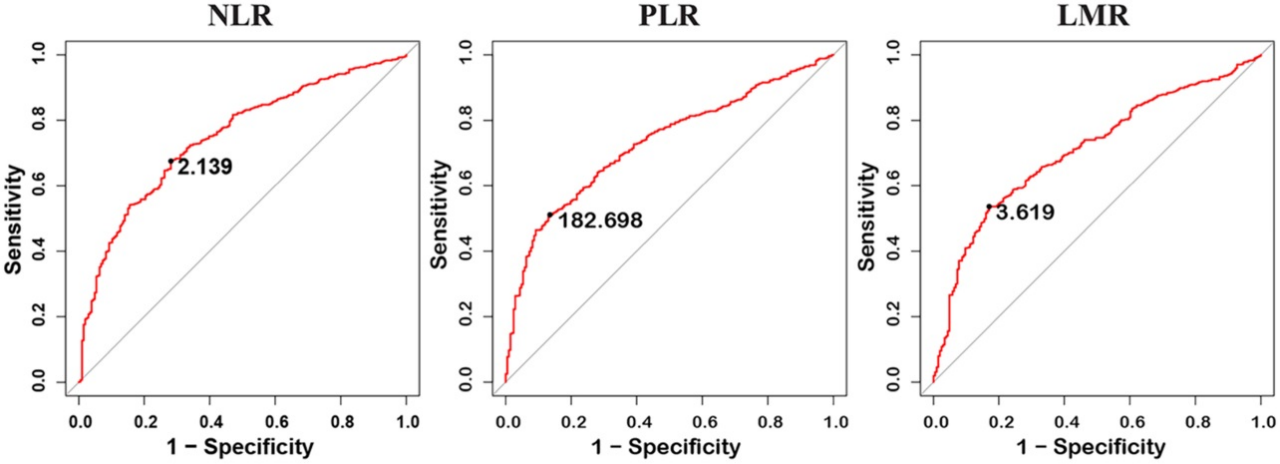
Receiver operating characteristic (ROC) curves analysis of neutrophil-lymphocyte ratio (NLR), platelet-lymphocyte ratio (PLR), and lymphocyte-monocyte ratio (LMR) for discriminating between malignant ovarian cancer and benign ovarian masses.

The optimal cut-off points based on ROC curve analyses for NLR, PLR, and LMR were found to be 2.139 (sensitivity 0.676, specificity 0.718), 182.698 (sensitivity 0.511, specificity 0.865) and 3.619 (sensitivity 0.536, specificity 0.830), respectively (Figure 1). For CA125, levels higher than cut-off value (35UI/L) had the 89.5% sensitivity and 70.9% specificity for the diagnosis of EOC.

**Figure 2:**
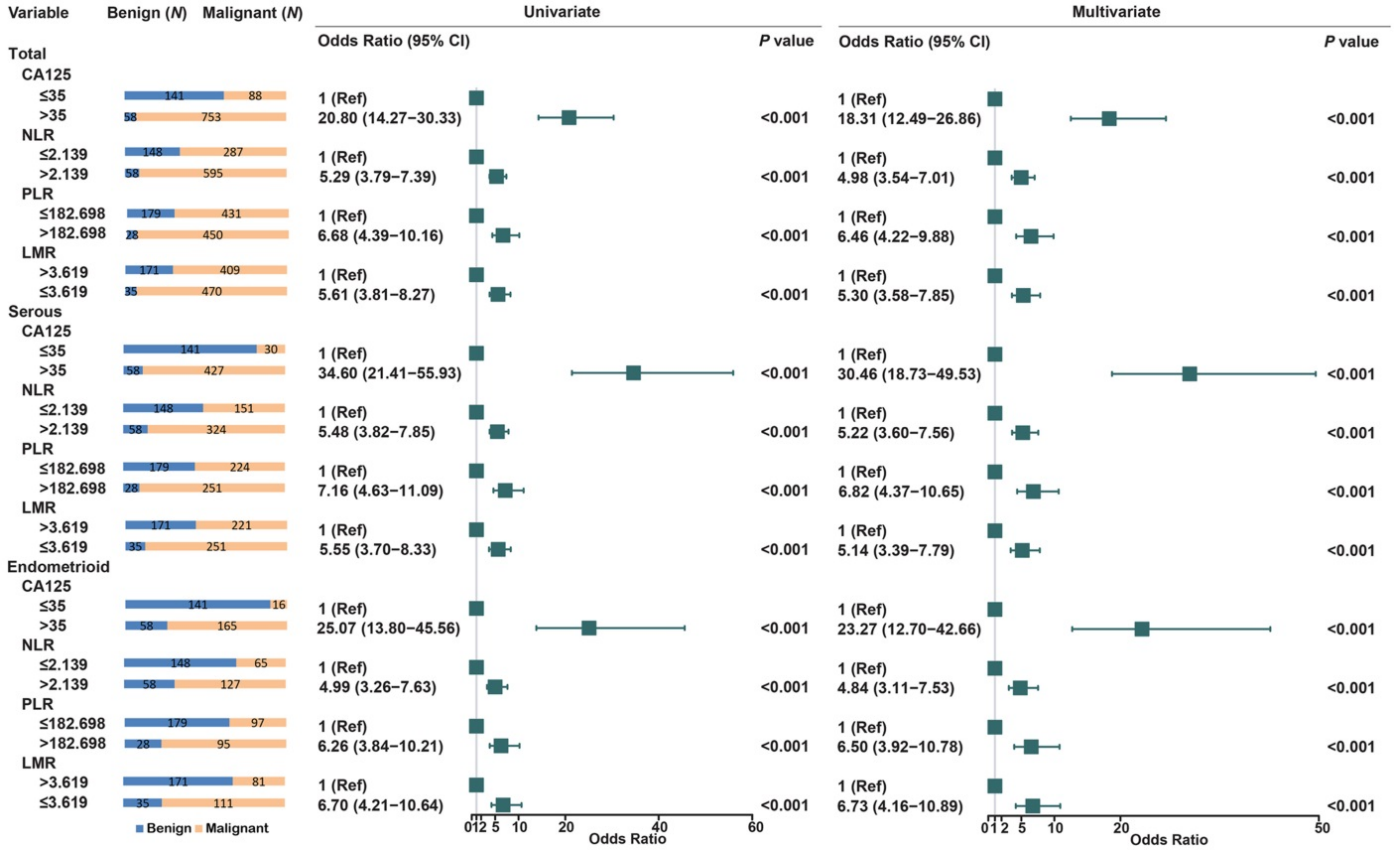
Univariate and multivariate analyses for EOC risk in stratified analysis by histological subtypes.

We further assessed the association between these biomarkers and EOC risk (Figure 2). Compared to CA125≤35, CA125>35 have increased the risk of EOC nearly 20-fold (OR =20.80, 95% CI: 14.27-30.33). High levels of NLR (OR = 5.29, 95% CI: 3.79-7.39) and PLR (OR = 6.68, 95% CI: 4.39-10.16) were also significantly associated with EOC risk. LMR≤3.619 increase the risk of EOC (OR = 5.61, 95% CI: 3.81-8.27), when compared to LMR>3.619. Histological stratified analyses showed that NLR, PLR and LMR were also increase the risk of serous, endometrioid, and other types of EOC.

**Figure 3:**
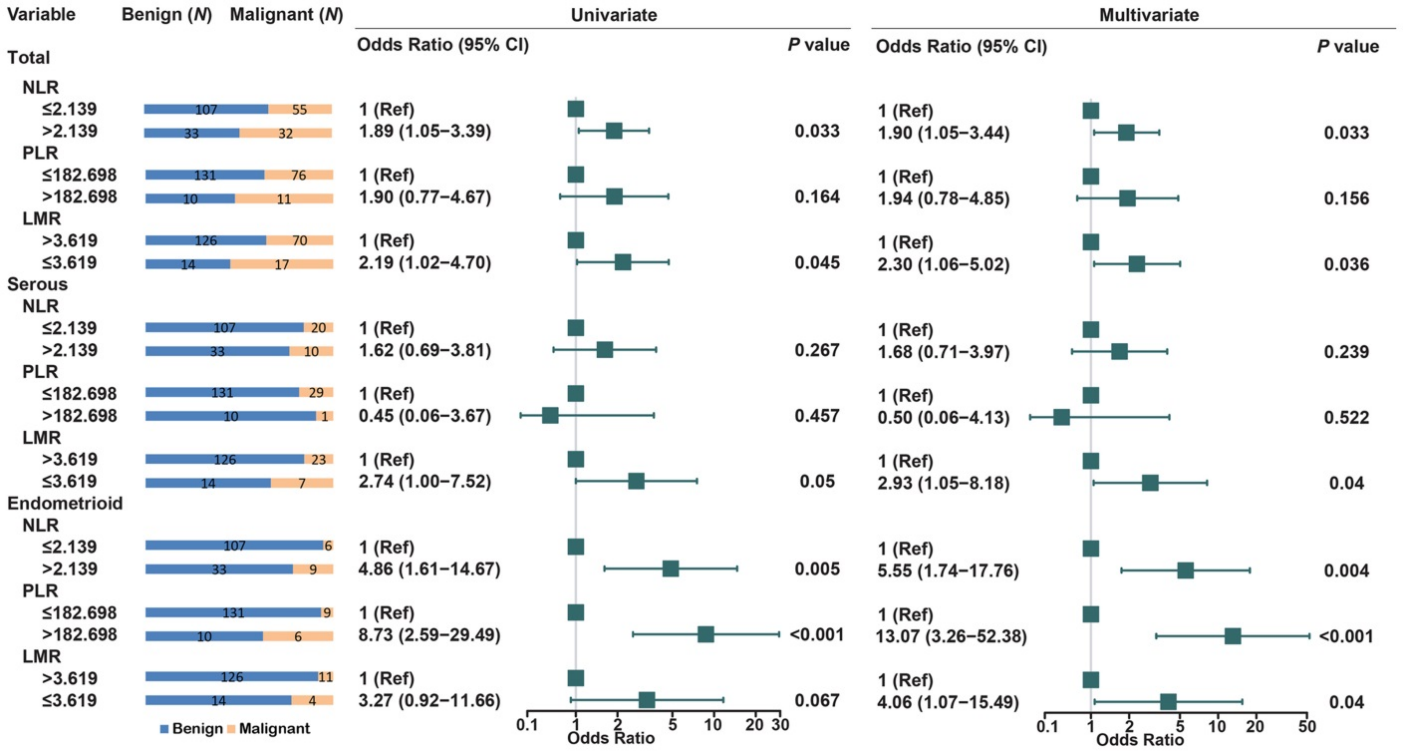
Univariate and multivariate analyses for EOC risk in stratified analysis by histogical subtypes in low level of CA125 patients (CA125≤35).

Of 841 EOC patients, CA125 level was not elevated in 88 EOC patients (CA125≤35). Therefore, we compared the levels of NLR, PLR, and LMR between 141 benign masses and 88 EOC patients (CA125≤35). Of the three biomarkers, high level of NLR (OR = 1.89, 95% CI: 1.05-3.39) and low level of LMR (OR = 2.19, 95% CI: 1.02-4.70) was significantly associated with the risk of EOC. In stratified analysis by histology, only low level of LMR was significantly increased the risk of serous ovarian cancers (OR = 2.74, 95% CI: 1.00-7.52). High level of NLR and PLR increased the risk of endometrioid EOC (OR=4.86, 95% CI: 1.61-14.67; OR=8.73, 95% CI: 2.59-29.49) (Figure 3). In addition, NLR, PLR and LMR was associated with all stages and subtypes of EOC in 58 benign ovarian mass and 753 EOC with high CA125 level (CA125>35) (Figure S4- Figure S7).

## Discussion

In current study, we evaluated the utility of preoperative parameters of complete blood count to distinguish EOC from benign ovarian masses and proposed the optimal cutoff of NLR, PLR, and LMR to predict the presence of EOC. In addition, we showed the prediction performance in different pathology types of EOC for the first time.

CA125 is the most commonly used serological marker for ovarian cancer. A number of studies have demonstrated that CA125 can be used for the diagnosis of ovarian cancer 2, 5-8, 19, 22-27, which is consistent with our study. However, nearly 20% of patients of EOC have lower level of CA125, therefore, CA125 test alone should not be used to differentiate between a benign and a malignant adnexal mass 5, 10. In the present study, we found that NLR, PLR, and LMR, in combination with CA125 could contribute to predict the presence of EOC.

A number of studies have been reported that NLR, PLR, and LMR could be used as a diagnosis and prognosis marker of ovarian cancer patients 3, 17-20, 28-33. Cho et al. 17 found that the preoperative NLR of patients with ovarian cancer was significantly higher than that of patients with benign ovarian masses. The cut-off value based on ROC curve analysis was 2.60, and the sensitivity and specificity were 66.1% and 82.7%, respectively. Polat et al. found that the optimal cutoff value of the NLR was 2.47, with 63.4% sensitivity and 63.5% specificity for malignancy prediction 30. Wan et al. reported that preoperative NLR, with a cut-off value of 2.64, is an independent predictor of EOC 20. In present study, we reached similar results. The optimal cut-off value based on ROC curve analysis was 2.139, with a sensitivity of 71.8% and a specificity of 67.6% for ovarian cancer prediction. Raungkaewmanee et al. 34 found that PLR of 200 yielded the most optimal predictive value to predict advanced stage disease, and the AUC was 0.66 (95% CI, 0.59 to 0.73) while the sensitivity and specificity were 59.0% and 72.7%, respectively. Similar to our results, we evaluated the predictive value of PLR in the diagnosis of ovarian cancer and found a PLR of 182.698 yielded a good predictive value. However, the PLR had an AUC of 0.730, with the highest specificity among other blood parameters but with relatively lower sensitivity. Previous studies have demonstrated that low level of LMR was an independent risk factors for progression-free survival (PFS) and overall survival (OS) in EOC patients, and the cut off values of these studies ranged from 2.07 to 3.84 31, 35, 36. Eo et al. 20 identified LMR as a predictor of the presence of EOC in patients with an ovarian mass (OR = 0.51, P = 0.024). Preoperative LMR with a cut-off value of 3.52 predicted the presence of EOC. In our study, the cut-off value for LMR was 3.619, by the ROC curve analysis, is an independent predictive factor in EOC patients. We also found that NLR, PLR, and LMR could be a predictor of the presence of EOC in all stage and subtypes of EOC.

In current study, using the cut-off value of CA125 level of 35UI/L, 11.7% (88 of 841 EOC) of EOC were still missed. We found that high level of NLR and PLR with above optimal cutoff values increase the risk of endometrioid EOC, while low level of LMR were significantly associated with an increased risk of serous EOC in low CA125 level patients. This suggested that NLR, PLR, and LMR could be applied together with CA125 to predict the presence of EOC as well as the histological subtype of EOC in low CA125 level patients. It is reported that the frequency of elevated NLR (NLR≥4) was highest in patients with serous EOC followed by clear cell, endometrioid, and mucinous types and the significant difference was observed between high and low NLR group in different histological types of EOC 37. The diagnostic accuracy of inflammatory markers for distinguishing benign ovarian masses and malignant may be different according to histologic subtypes. However, this study lacked external validation of the main findings and further validation studies in an independent cohort are warranted. In addition, this was a retrospective study, a prospective study with large sample size would be needed to apply as a screening tool for ovarian cancer.

## Conclusions

The findings of this study suggested that in addition to CA125 biomarkers, NLR, PLR and LMR could be used as predictors of EOC. We also found that preoperative inflammation markers may be a potential predictive marker of histology subtypes of EOC, which need to be validated by additional well-designed study.

## Supplementary Material

Supplementary figures and tables.

## Figures and Tables

**Table 1:** Comparison of variables between benign ovarian masses and malignant ovarian masses groups.

	Benign ovarian masses		Malignant ovarian cancer		*P* value
	*N*	Median (IQR)	*N*	Median (IQR)	
CA125 (U/mL)	199	17.91 (36.0)	841	606.0 (1221.7)	<0.001
HGB (g/L)	207	130.0 (14.0)	885	126.0 (17.0)	<0.001
MCV (Fl)	207	90.5 (5.4)	885	89.4 (5.9)	0.005
HCT (%)	207	39.3 (4.3)	884	38.6 (4.3)	0.002
WBC (10^^^9/L)	206	5.76 (2.13)	884	6.55 (2.29)	<0.001
NLR	206	1.68 (1.01)	883	2.61 (1.83)	<0.001
PLR	207	131.0 (59.1)	882	184.6 (122.6)	<0.001
LMR	206	4.87 (2.30)	881	3.50 (2.58)	<0.001

CA125, Cancer Antigen 125; HGB, hemoglobin concentration; MCV, mean corpuscular volume; HCT, hematocrit; WBC, white blood cell count; NLR, neutrophil-to-lymphocyte ratio; PLR, platelet-to-lymphocyte ratio; LMR, lymphocyte lymphocyte-to-monocyte ratio; IQR, interquartile range.

**Table 2:** Comparison of variables between benign ovarian masses and malignant ovarian masses of different pathological types.

	Benign ovarian masses		Serous			Endometrioid			Others		
	*N*	Median (IQR)	*N*	Median (IQR)	P^*^	*N*	Median (IQR)	P^*^	*N*	Median (IQR)	P^*^
CA125 (U/mL)	199	17.91 (36.0)	457	748.0 (1333.3)	<0.001	181	568.4 (1110.6)	<0.001	203	272.6 (952.4)	<0.001
HGB (g/L)	207	130.0 (14.0)	476	126.0 (17.0)	<0.001	194	126.0 (16.8)	0.001	215	126.0 (18.0)	0.001
MCV (Fl)	207	90.5 (5.4)	476	89.4 (5.8)	0.006	194	89.0 (5.6)	0.005	215	90.0 (6.2)	0.196
HCT (%)	207	39.3 (4.3)	475	38.5 (4.4)	0.002	194	38.7 (4.2)	0.055	215	38.8 (4.3)	0.022
WBC (10^^^9/L)	206	5.76 (2.13)	475	6.48 (2.13)	<0.001	194	6.79 (2.48)	<0.001	215	6.52 (2.28)	<0.001
NLR	206	1.68 (1.01)	475	2.56 (1.73)	<0.001	192	2.72 (1.77)	<0.001	215	2.60 (2.26)	<0.001
PLR	207	131.0 (59.1)	475	187.5 (133.0)	<0.001	192	180.0 (89.3)	<0.001	214	180.7 (149.3)	<0.001
LMR	206	4.87 (2.30)	472	3.51 (2.29)	<0.001	192	3.35 (2.32)	<0.001	215	3.61 (3.04)	<0.001

CA125, Cancer Antigen 125; HGB, hemoglobin concentration; MCV, mean corpuscular volume; HCT, hematocrit; WBC, white blood cell count; NLR, neutrophil-to-lymphocyte ratio; PLR, platelet-to-lymphocyte ratio; LMR, lymphocyte lymphocyte-to-monocyte ratio; IQR, interquartile range. ^*^ *P* value for each group compared with benign ovarian masses.

**Table 3:** Area under the curve (AUC) for discriminating all malignant cases from benign cases.

Variables	AUC	95% CI	Cut-off	Sensitivity (%)	Specificity (%)
CA125 (U/mL)	90.6	88.5-92.8	35	89.5	70.9
HGB (g/L)	59.9	55.7-64.0	124.5	45.1	71.0
MCV (Fl)	56.2	51.9-60.6	87.15	31.0	80.7
HCT (%)	56.8	52.5-61.0	38.95	54.9	57.0
WBC (10^^^9/L)	63.6	59.4-67.8	5.815	69.5	52.4
NLR	74.9	71.4-78.4	2.139	67.6	71.8
PLR	73.0	69.6-76.4	182.698	51.1	86.5
LMR	70.9	67.2-74.5	3.619	53.6	83.0

CA125, Cancer Antigen 125; HGB, hemoglobin concentration; MCV, mean corpuscular volume; HCT, hematocrit; WBC, white blood cell count; NLR, neutrophil-to-lymphocyte ratio; PLR, platelet-to-lymphocyte ratio; LMR, lymphocyte-to-monocyte ratio.
