# Supplementary material for: Utility of Preoperative Inflammatory Markers to Distinguish Epithelial Ovarian Cancer from Benign Ovarian Masses
Source: J Cancer. 2021 Mar 5;12(9):2687–93. doi: 10.7150/jca.51642 (PMC8040725; doi:10.7150/jca.51642)

Utility of Preoperative Inflammatory Markers to Distinguish Epithelial Ovarian Cancer from Benign Ovarian Masses

Lian Li [1,*], Jing Tian [1,2*], Liwen Zhang [1,*], Luyang Liu [1], Chao Sheng [1], Yubei Huang [1], Hong Zheng [1], Fengju Song [1], Kexin Chen [1]

[1] Department of Epidemiology and Biostatistics, National Clinical Research Center for Cancer, Key Laboratory of Cancer Prevention and Therapy of Tianjin, Tianjin Medical University Cancer Institute and Hospital, Tianjin, China.

[2] Department of Urology, National Clinical Research Center for Cancer, Key Laboratory of Cancer Prevention and Therapy of Tianjin, Tianjin Medical University Cancer Institute and Hospital, Tianjin, China.

Correspondence to: Kexin Chen, M.D., Ph.D., Professor and Chair, Department of Epidemiology and Biostatistics, National Clinical Research Center for Cancer, Key Laboratory of Cancer Prevention and Therapy of Tianjin, Tianjin Medical University Cancer Institute and Hospital, Huanhu Xi Road, Tiyuan Bei, Hexi District, Tianjin, P.R. China 300060. Tel.: ++86 (0)2223372231; Fax: ++86 (0)2223372231; E-mail: chenkexin@tjmuch.com.

* These authors contributed equally to this work.

Supplemental Figure Legends

Figure S1. Receiver operating characteristic (ROC) curves of inflammation markers in predicting risk of ovarian cancer by stage, (A) Early stages versus benign masses, (B) Advanced stages versus benign masses.

Figure S2. Receiver operating characteristic (ROC) curves of inflammation markers in predicting risk of ovarian cancer by histological subtype, (A) Serous ovarian cancer versus benign masses, (B) Endometrioid ovarian cancer versus benign masses, (C) Other types of EOC versus benign masses.

Figure S3. Receiver operating characteristic (ROC) curves of inflammation markers in predicting risk of ovarian cancer by menopausal status, (A) Premenopausal ovarian cancer versus benign masses, (B) Postmenopausal ovarian cancer versus benign masses.

Figure S4. Univariate and multivariate analyses for EOC risk in stratified analysis by histological subtypes in high level of CA125 patients (CA125>35).

Figure S5. Univariate and multivariate analyses for EOC risk in stratified analysis by stage.

Figure S6. Univariate and multivariate analyses for EOC risk in stratified analysis by stage in low level of CA125 patients (CA125≤35).

Figure S7. Univariate and multivariate analyses for EOC risk in stratified analysis by stage in high level of CA125 patients (CA125>35).

SUPPLEMENTAL TABLES

Table S1. Demographic and epidemiological characteristics of patients.

	Benign (*N*=207, *N*, %)	Malignant (*N*=887, *N*, %)	*P* value
Age (years, mean±SD)	47.17±14.66	54.23± 10.57	<0.001
Menopause			
No	106 (55.50)	280 (33.14)	<0.001
Yes	85 (44.50)	565 (66.86)	
BMI (kg/m^2)			
<25	127 (61.35)	571 (64.37)	0.415
≥25	80 (38.65)	316 (35.63)	
Family history of cancer			
No	161 (81.31)	649 (75.47)	0.080
Yes	37 (18.69)	211 (24.53)	
Alcohol consumption			
No	196 (99.49)	824 (97.51)	0.044
Yes	1 (0.51)	21 (2.49)	
Age of menarche (years)			
≤13	64 (30.92)	231 (26.04)	0.155
>13	143 (69.08)	656 (73.96)	
Number of pregnancies			
≤2	104 (50.24)	425 (47.91)	0.546
>2	103 (49.76)	462 (52.09)	
Number of live births			
≤1	133 (64.25)	518 (58.40)	0.123
>1	74 (35.75)	369 (41.60)	
Abortion			
No	58 (28.02)	295 (33.26)	0.147
Yes	149 (71.98)	592 (66.74)	

BMI, body mass index

Table S2. Clinical and pathological features of patients.

	Benign ovarian masses (*N*=207, *N*, %)	Malignant ovarian cancer (*N*=887, *N*, %)
Tumor diameter (cm)		
<5	85 (41.06)	337 (37.99)
≥5	122 (58.94)	550 (62.01)
Pathology		
Benign ovarian masses		
Serous cystadenoma	33 (15.94)	—
Mucinous cystadenoma	11 (5.31)	—
Mature teratoma	81 (39.13)	—
Others	82(39.62)	—
Epithelial ovarian cancer		
Serous	—	477 (53.78)
Mucinous	—	55 (6.20)
Endometrioid	—	194 (21.87)
Other EOC	—	161 (18.15)
Stage		
I	—	133 (15.36)
II	—	165 (19.05)
III	—	483 (55.77)
IV	—	85 (9.82)
Residual disease (cm)		
≤1	—	437 (75.22)
>1	—	144 (24.78)

EOC, epithelial ovarian cancer

Table S3. Univariate and multivariate logistic regression model showing association of biomarkers with malignancy.

Variables	OR (95%CI)	*P* value	OR (95%CI)[*]	*P* value [*]
CA125 (U/mL)	1.006 (1.005-1.007)	<0.001	1.006 (1.004-1.007)	<0.001
HGB (g/L)	0.972 (0.960-0.985)	<0.001	0.973 (0.960-0.986)	<0.001
MCV (Fl)	0.964 (0.934-0.995)	0.024	0.946 (0.914-0.978)	0.001
HCT (%)	0.931 (0.889-0.974)	0.002	0.928 (0.884-0.973)	0.002
WBC (10^9/L)	1.310 (1.191-1.447)	<0.001	1.323 (1.199-1.467)	<0.001
NLR	2.061 (1.750-2.455)	<0.001	2.009 (1.701-2.340)	<0.001
PLR	1.012 (1.009-1.014)	<0.001	1.011 (1.009-1.014)	<0.001
LMR	0.811 (0.756-0.868)	<0.001	0.823 (0.766-0.883)	<0.001
Age at diagnosis	1.054 (1.039-1.068)	<0.001	-	-

CA125, Cancer Antigen 125; HGB, hemoglobin; MCV, mean corpuscular volume; HCT, hematocrit; WBC, white blood cell count; NLR, neutrophil-to-lymphocyte ratio; PLR, platelet-to-lymphocyte ratio; LMR, lymphocyte lymphocyte-to- monocyte ratio; OR = odds ratio, CI = confidence interval.

[*] adjusted for age.

Table S4. Area under the curve (AUC) for discriminating all malignant cases from benign cases adjusted for age.

Variables	AUC	95% CI	Cut-off	Sensitivity (%)	Specificity (%)
CA125 (U/mL)	90.8	88.6-92.9	193.65	78.6	89.4
HGB (g/L)	54.2	49.5-58.9	165.5	79.5	32.4
MCV (Fl)	62.4	57.7-67.2	130.35	86.2	38.6
HCT (%)	62.8	58.0-67.6	83.55	80.8	45.9
WBC (10^9/L)	67.4	62.8-72.1	53.525	78.8	55.8
NLR	67.6	62.9-72.3	49.673	78.6	54.4
PLR	75.4	72.1-78.8	215.023	62.2	78.3
LMR	62.7	57.9-67.5	52.835	70.2	56.3

CA125, Cancer Antigen 125; HGB, hemoglobin; MCV, mean corpuscular volume; HCT, hematocrit; WBC, white blood cell count; NLR, neutrophil-to-lymphocyte ratio; PLR, platelet-to-lymphocyte ratio; LMR, lymphocyte lymphocyte-to- monocyte ratio; AUC, Area under curve; CI = confidence interval.

Table S5. Area under the curve (AUC) for discriminating malignant from benign in stratified analysis by stage.

Variables	AUC	95% CI	Cut-off	Sensitivity (%)	Specificity (%)
Early stage malignant vs. benign					
CA125 (U/mL)	81.3	77.6-85.1	59.03	68.4	78.9
HGB (g/L)	54.7	49.6-59.8	131.5	62.8	46.9
MCV (Fl)	51.2	46.0-56.3	87.15	23.8	80.7
HCT (%)	52.4	47.3-57.6	42.15	87.6	18.4
WBC (10^9/L)	61.9	56.9-66.9	5.295	80.9	40.3
NLR	62.3	57.4-67.2	2.015	53.4	66.5
PLR	58.3	53.3-63.3	141.864	56.0	60.9
LMR	56.5	51.5-61.6	3.619	32.8	83.0
Advantaged stage malignant vs. benign					
CA125 (U/mL)	95.4	93.6-97.1	228.7	85.2	95.5
HGB (g/L)	62.6	58.2-66.9	123.5	47.9	72.9
MCV (Fl)	58.6	54.1-63.2	87.15	34.5	80.7
HCT (%)	59.0	54.6-63.5	38.75	56.3	59.9
WBC (10^9/L)	64.4	60.1-68.8	5.815	71.0	52.4

NLR	81.6	78.2-84.9	2.139	78.3	71.8
PLR	80.9	77.7-84.1	196.41	60.3	90.8
LMR	78.5	74.9-82.0	3.615	65.0	83.0

CA125, Cancer Antigen 125; HGB, hemoglobin; MCV, mean corpuscular volume; HCT, hematocrit; WBC, white blood cell count; NLR, neutrophil-to-lymphocyte ratio; PLR, platelet-to-lymphocyte ratio; LMR, lymphocyte lymphocyte-to- monocyte ratio; AUC, Area under curve; CI = confidence interval.

Table S6. Area under the curve (AUC) for discriminating malignant from benign in stratified analysis by stage adjusted for age.

Variables	AUC	95%CI	Cut-off	Sensitivity (%)	Specificity (%)
Early stage malignant vs. benign					
CA125 (U/mL)	81.1	77.2-84.9	106.535	70.9	77.4
HGB (g/L)	55.3	50.2-60.5	164.5	80.9	30.4
MCV (Fl)	60.8	55.7-65.9	130.35	82.9	38.6
HCT (%)	60.4	55.3-65.6	83.55	76.2	45.9
WBC (10^9/L)	63.7	58.6-68.8	53.56	72.1	55.8
NLR	62.8	57.6-67.9	51.023	66.4	57.8
PLR	61.4	56.5-66.4	192.133	56.7	62.3
LMR	60.9	55.7-66.1	52.874	66.2	56.3
Advantaged stage malignant vs. benign					
CA125 (U/mL)	95.7	94.1-97.4	276.4	85.4	96.0
HGB (g/L)	53.6	48.6-58.5	162.5	84.8	27.5
MCV (Fl)	63.3	58.4-68.2	131.25	86.7	40.1
HCT (%)	64.1	59.1-69.0	83.6	83.2	45.9
WBC (10^9/L)	69.4	64.6-74.2	53.525	82.3	55.8

NLR	70.1	65.3-74.9	49.673	83.8	54.4
PLR	83.0	79.8-86.1	215.385	74.9	78.3
LMR	63.7	58.6-68.7	52.835	72.3	56.3

CA125, Cancer Antigen 125; HGB, hemoglobin; MCV, mean corpuscular volume; HCT, hematocrit; WBC, white blood cell count; NLR, neutrophil-to-lymphocyte ratio; PLR, platelet-to-lymphocyte ratio; LMR, lymphocyte lymphocyte-to- monocyte ratio; AUC, Area under curve; CI = confidence interval.

Table S7. Area under the curve (AUC) for discriminating malignant from benign in stratified analysis by histological subtype.

Variables	AUC	95%CI	Cut-off	Sensitivity	Specificity
Serous ovarian cancer vs. benign					
CA125 (U/mL)	93.1	91.0-95.2	228.7	78.8	95.5
HGB (g/L)	60.5	55.9-65.0	124.5	46.0	71.0
MCV (Fl)	56.6	51.9-61.4	87.15	31.7	80.7
HCT (%)	57.4	52.8-62.0	38.65	52.4	60.9
WBC (10^9/L)	62.0	57.8-66.9	5.815	69.7	52.4
NLR	74.7	70.8-78.6	2.14	68.2	71.8
PLR	73.6	69.8-77.4	182.698	52.8	86.5
LMR	71.2	67.1-75.3	3.619	53.4	83.0
Endometrioid ovarian cancer vs. benign					
CA125 (U/mL)	91.7	88.8-94.5	129.0	84.0	88.4
HGB (g/L)	59.3	53.8-64.9	124.5	45.4	71.0
MCV (Fl)	58.2	52.6-63.7	90.05	60.8	54.6
HCT (%)	55.5	49.9-61.2	38.75	51.5	59.9
WBC (10^9/L)	66.5	61.3-71.8	7.14	42.8	80.6

NLR	76.1	71.4-80.7	2.49	57.8	84.5
PLR	73.0	68.0-78.0	155.814	67.2	71.5
LMR	73.5	68.6-78.5	3.599	57.8	83.0
Other EOC vs. benign					
CA125 (U/mL)	84.1	80.3-87.9	59.03	74.4	78.9
HGB (g/L)	59.0	53.6-64.5	129.5	62.3	53.1
MCV (Fl)	53.6	48.1-59.2	87.15	29.3	80.7
HCT (%)	56.5	51.0-61.9	35.65	21.4	89.9
WBC (10^9/L)	63.7	58.5-69.0	5.3	83.3	40.3
NLR	74.3	69.7-79.0	2.139	67.4	71.8
PLR	71.7	66.7-76.6	196.477	45.3	90.8
LMR	67.8	62.6-72.9	3.615	50.2	83.0

CA125, Cancer Antigen 125; HGB, hemoglobin; MCV, mean corpuscular volume; HCT, hematocrit; WBC, white blood cell count; NLR, neutrophil-to-lymphocyte ratio; PLR, platelet-to-lymphocyte ratio; LMR, lymphocyte lymphocyte-to- monocyte ratio; EOC, epithelial ovarian cancer; AUC, Area under curve; CI = confidence interval.

Table S8. Area under the curve (AUC) for discriminating malignant from benign in stratified analysis by histological subtype adjusted for age.

Variables	AUC	95%CI	Cut-off	Sensitivity	Specificity
Serous ovarian cancer vs. benign					
CA125 (U/mL)	93.3	91.3-95.4	276.4	78.8	96.0
HGB (g/L)	54.0	49.0-59.0	164.5	83.6	30.4
MCV (Fl)	62.8	57.8-67.7	131.25	85.5	40.1
HCT (%)	63.2	58.1-68.2	83.55	82.7	45.9
WBC (10^9/L)	67.8	62.9-72.7	53.525	80.6	55.8
NLR	68.0	63.1-72.9	49.949	79.2	54.9
PLR	76.1	72.4-79.8	212.59	64.0	77.3
LMR	63.3	58.2-68.3	52.835	72.5	56.3
Endometrioid ovarian cancer vs. benign					
CA125 (U/mL)	92.4	89.7-95.1	203.15	84.0	89.9
HGB (g/L)	55.1	49.5-60.8	173.5	68.6	44.4
MCV (Fl)	62.2	56.7-67.7	129.75	89.7	37.2
HCT (%)	63.4	58.0-68.9	77.7	93.3	33.8
WBC (10^9/L)	68.0	62.8-73.3	49.28	92.3	40.8

NLR	67.6	62.3-72.9	45.712	91.1	40.8
PLR	75.6	70.8-80.4	226.352	60.9	82.6
LMR	62.7	57.1-68.2	46.36	90.1	36.4
Other EOC vs. benign					
CA125 (U/mL)	83.5	79.6-87.5	106.535	76.4	77.4
HGB (g/L)	53.7	48.1-59.2	172.5	67.9	42.5
MCV (Fl)	61.9	56.5-67.3	138.75	67.9	57.0
HCT (%)	61.6	56.2-67.0	78.25	87.4	35.3
WBC (10^9/L)	66.2	60.9-71.4	53.24	79.1	53.9
NLR	66.6	61.4-71.9	49.707	78.6	54.4
PLR	74.0	69.3-78.7	261.421	44.9	93.2
LMR	61.5	56.1-67.0	53.275	67.0	57.8

CA125, Cancer Antigen 125; HGB, hemoglobin; MCV, mean corpuscular volume; HCT, hematocrit; WBC, white blood cell count; NLR, neutrophil-to-lymphocyte ratio; PLR, platelet-to-lymphocyte ratio; LMR, lymphocyte lymphocyte-to- monocyte ratio; EOC, epithelial ovarian cancer; AUC, Area under curve; CI = confidence interval.

Table S9. Area under the curve (AUC) for discriminating malignant from benign in stratified analysis by menopausal status.

Variables	AUC	95%CI	Cut-off	Sensitivity	Specificity
Premenopausal					
CA125 (U/mL)	89.5	86.0-93.0	228.7	71.3	94.1
HGB (g/L)	58.5	52.2-64.8	132.5	70.3	44.3
MCV (Fl)	56.3	50.0-62.6	87.25	40.9	74.5
HCT (%)	56.2	49.8-62.6	35.95	26.9	84.9
WBC (10^9/L)	63.7	57.6-69.8	5.81	74.5	47.2
NLR	74.9	69.8-80.0	2.59	55.2	84.0
PLR	74.7	69.7-79.7	182.243	54.8	87.7
LMR	73.6	68.5-78.6	3.624	56.6	84.9
Postmenopausal					
CA125 (U/mL)	90.9	88.0-93.9	133.95	78.8	91.5
HGB (g/L)	61.7	55.7-67.6	123.5	41.7	81.2
MCV (Fl)	60.0	53.4-66.6	93.35	81.6	35.3
HCT (%)	58.8	52.6-65.0	38.75	49.9	68.2
WBC (10^9/L)	64.4	57.9-70.9	6.030	61.3	65.5

NLR	75.0	69.6-80.4	2.113	66.9	73.8
PLR	71.5	66.1-76.8	196.197	45.1	90.6
LMR	69.1	63.2-75.0	3.721	52.9	81.0

CA125, Cancer Antigen 125; HGB, hemoglobin; MCV, mean corpuscular volume; HCT, hematocrit; WBC, white blood cell count; NLR, neutrophil-to-lymphocyte ratio; PLR, platelet-to-lymphocyte ratio; LMR, lymphocyte lymphocyte-to- monocyte ratio; AUC, Area under curve; CI = confidence interval.

Table S10. Area under the curve (AUC) for discriminating malignant from benign in stratified analysis by menopausal status adjusted for age.

Variables	AUC	95%CI	Cut-off	Sensitivity	Specificity
Premenopausal					
CA125 (U/mL)	89.2	85.6-92.7	275.05	70.9	95.0
HGB (g/L)	55.4	48.9-61.9	172.5	43.4	68.9
MCV (Fl)	65.8	59.6-72.1	129.9	65.9	61.3
HCT (%)	67.2	60.9-73.6	77.4	78.5	54.7
WBC (10^9/L)	74.7	69.2-80.2	47.225	79.1	59.4
NLR	74.8	69.3-80.4	43.012	80.6	60.4
PLR	76.8	72.0-81.7	224.952	54.8	89.6
LMR	67.1	60.9-73.3	45.159	74.2	56.6
Postmenopausal					
CA125 (U/mL)	90.3	87.3-93.3	190.75	79.1	90.2
HGB (g/L)	63.3	57.1-69.5	190.5	63.8	55.3
MCV (Fl)	57.6	50.9-64.3	150.55	59.8	55.3
HCT (%)	57.7	50.9-64.5	97.05	49.7	64.7
WBC (10^9/L)	51.2	44.1-58.3	65.115	51.4	57.1

NLR	50.5	43.3-57.7	73.529	90.0	17.9
PLR	71.3	65.9-76.7	209.7	66.5	69.4
LMR	58.9	52.0-65.9	66.898	71.1	46.4

HGB, hemoglobin; MCV, mean corpuscular volume; HCT, hematocrit; WBC, white blood cell count; PLT, platelet; NP, neutrophil percentage; LP, lymphocyte percentage; NLR, neutrophil-to-lymphocyte ratio; PLR, platelet-to-lymphocyte ratio; AUC, Area under curve; CI = confidence interval.

SUPPLEMENTAL FIGURES

Figure S1.

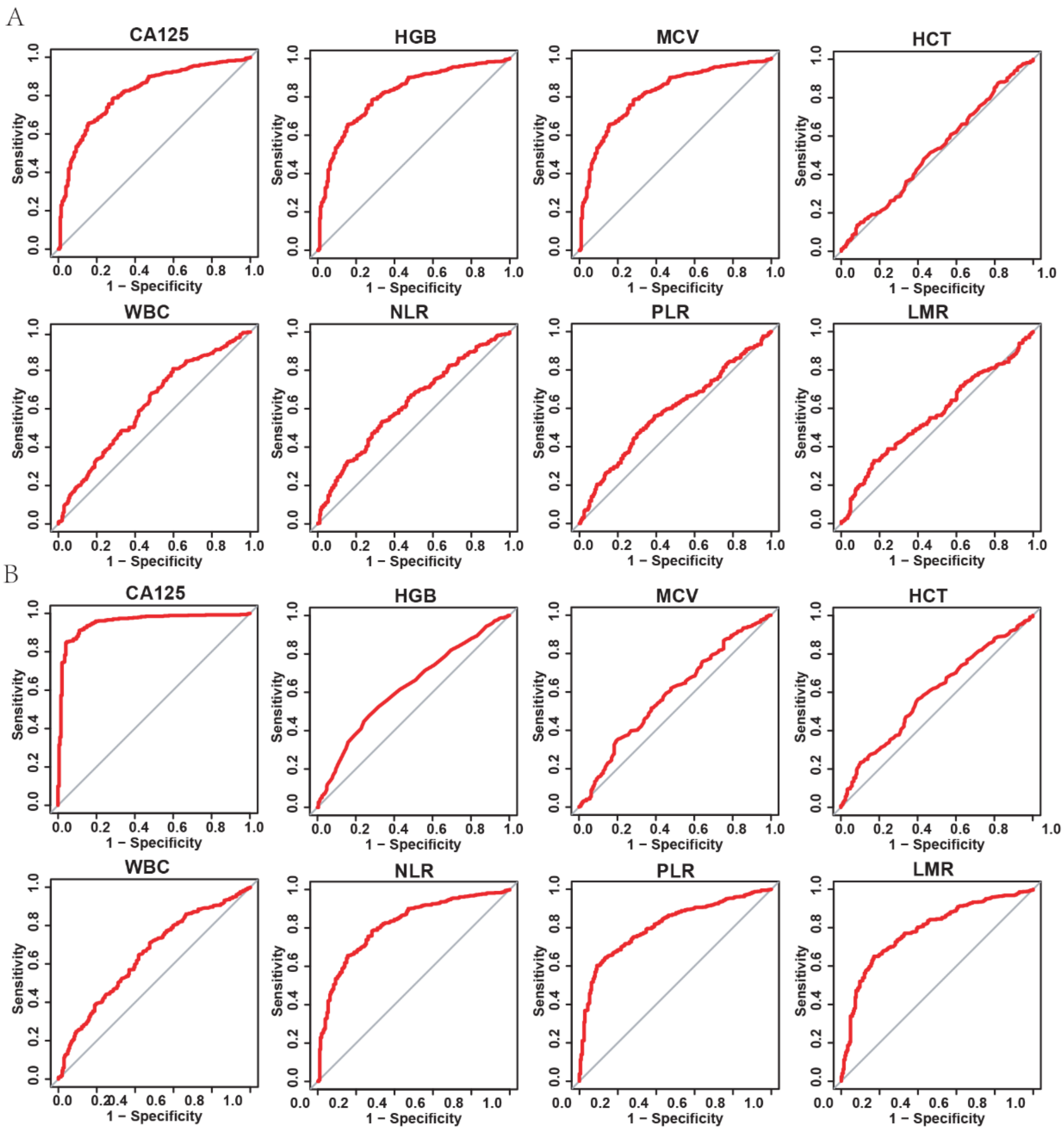

Figure S2.

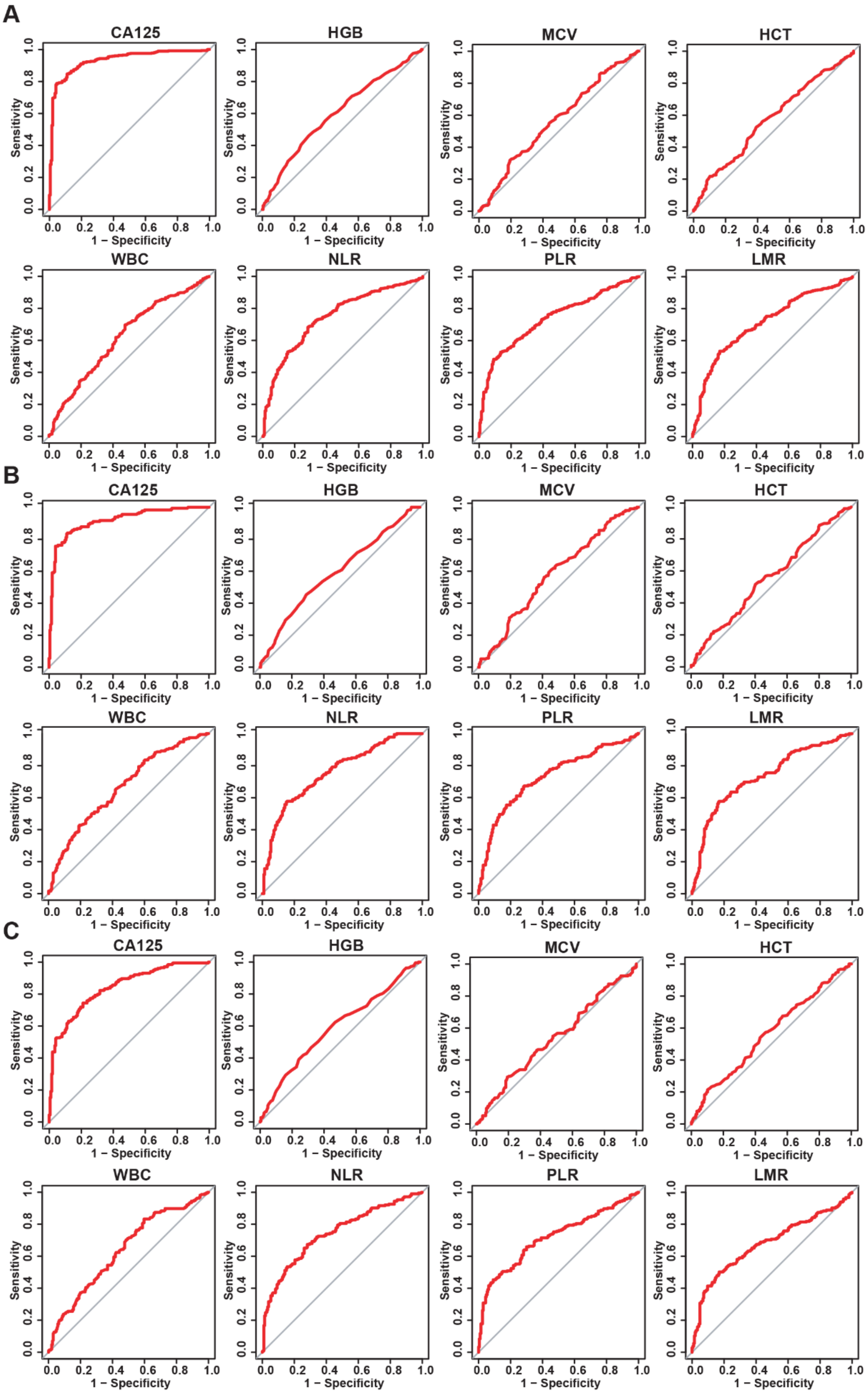
A
CA125
HGB
MCV
HCT
WBC
NLR
PLR
LMR
B
CA125
HGB
MCV
HCT
WBC
NLR
PLR
LMR
C
CA125
HGB
MCV
HCT
WBC
NLR
PLR
LMR
Sensitivity
1 − Specificity

Figure S3.

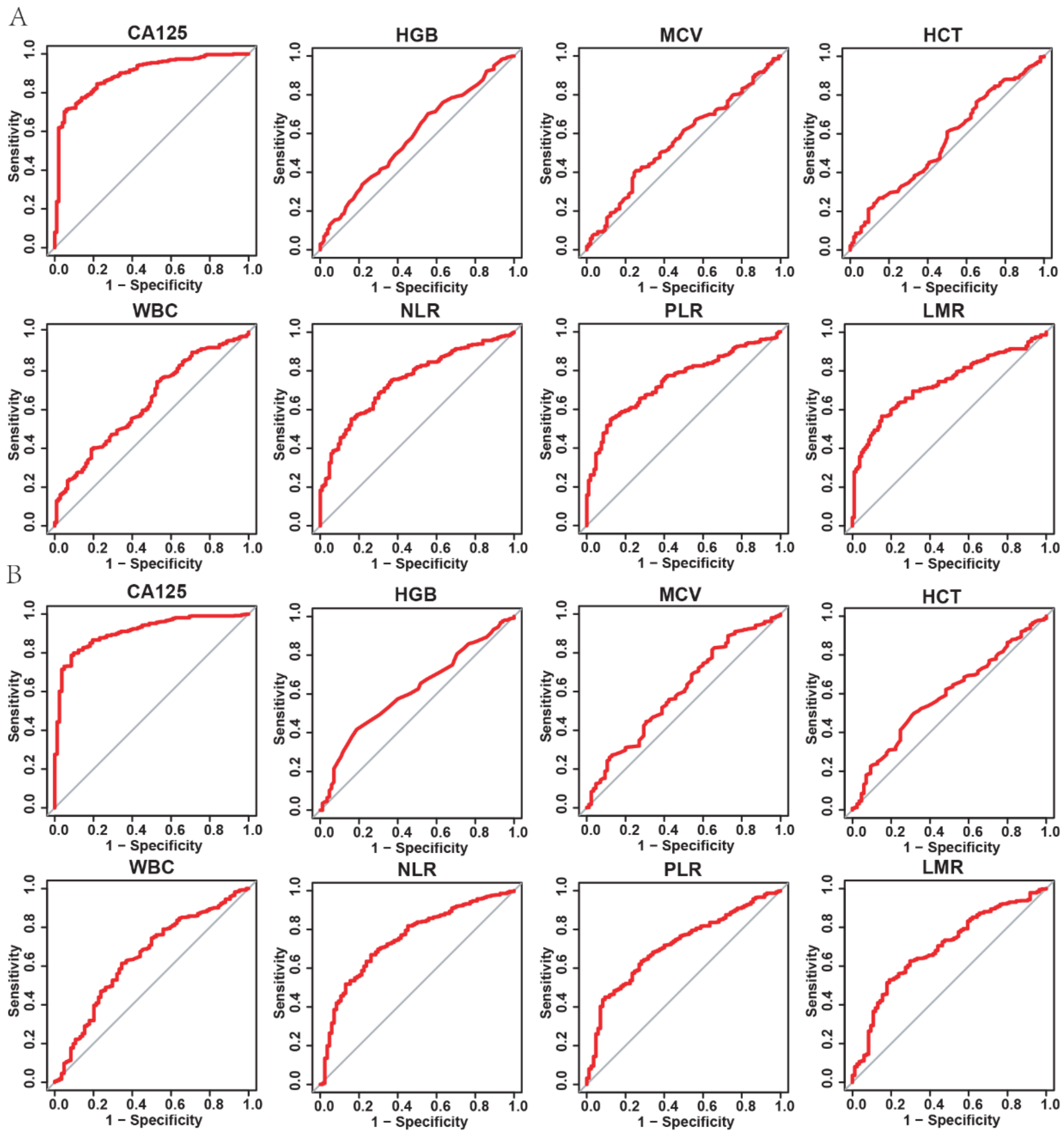

A
CA125
HGB
MCV
HCT
WBC
NLR
PLR
LMR
B
CA125
HGB
MCV
HCT
WBC
NLR
PLR
LMR
Sensitivity
1 – Specificity
0.0
0.2
0.4
0.6
0.8
1.0

Figure S4.

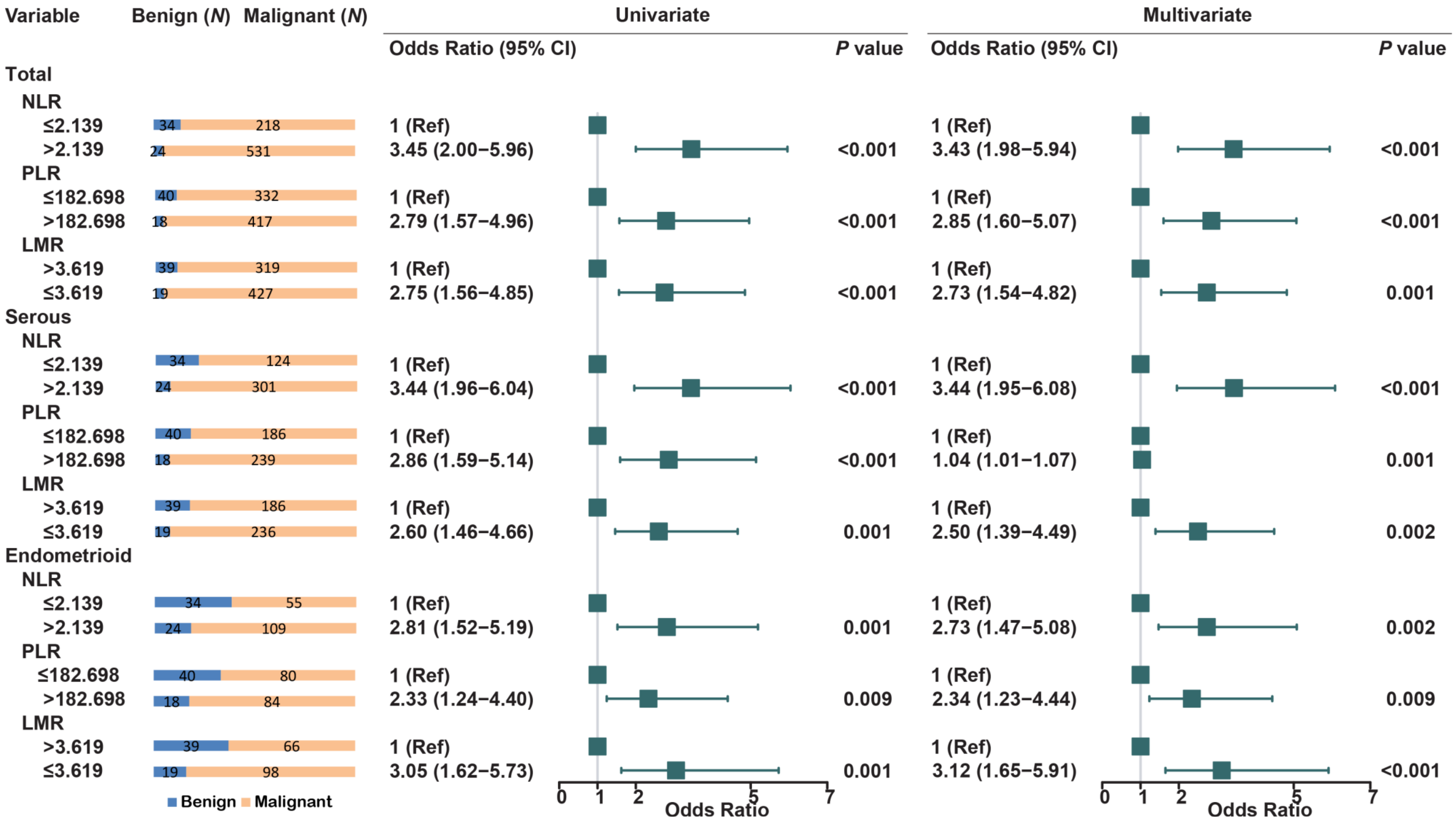
Variable
Benign (N)
Malignant (N)
Univariate
Multivariate
Odds Ratio (95% CI)
P value
Odds Ratio (95% CI)
P value
Total
NLR
≤2.139 34 218 1 (Ref) 1 (Ref)
>2.139 24 531 3.45 (2.00–5.96) <0.001 3.43 (1.98–5.94) <0.001
PLR
≤182.698 40 332 1 (Ref) 1 (Ref)
>182.698 18 417 2.79 (1.57–4.96) <0.001 2.85 (1.60–5.07) <0.001
LMR
>3.619 39 319 1 (Ref) 1 (Ref)
≤3.619 19 427 2.75 (1.56–4.85) <0.001 2.73 (1.54–4.82) 0.001
Serous
NLR
≤2.139 34 124 1 (Ref) 1 (Ref)
>2.139 24 301 3.44 (1.96–6.04) <0.001 3.44 (1.95–6.08) <0.001
PLR
≤182.698 40 186 1 (Ref) 1 (Ref)
>182.698 18 239 2.86 (1.59–5.14) <0.001 1.04 (1.01–1.07) 0.001
LMR
>3.619 39 186 1 (Ref) 1 (Ref)
≤3.619 19 236 2.60 (1.46–4.66) 0.001 2.50 (1.39–4.49) 0.002
Endometrioid
NLR
≤2.139 34 55 1 (Ref) 1 (Ref)
>2.139 24 109 2.81 (1.52–5.19) 0.001 2.73 (1.47–5.08) 0.002
PLR
≤182.698 40 80 1 (Ref) 1 (Ref)
>182.698 18 84 2.33 (1.24–4.40) 0.009 2.34 (1.23–4.44) 0.009
LMR
>3.619 39 66 1 (Ref) 1 (Ref)
≤3.619 19 98 3.05 (1.62–5.73) 0.001 3.12 (1.65–5.91) <0.001
Benign Malignant
0 1 2 5 7
Odds Ratio
0 1 2 5 7
Odds Ratio

Figure S5.

Variable	Benign (*N*)	Malignant (*N*)	Univariate Odds Ratio (95% CI)	Univariate *P* value	Multivariate Odds Ratio (95% CI)	Multivariate *P* value
**Total**						
CA125						
≤35	141	88	1 (Ref)		1 (Ref)	
>35	58	753	20.80 (14.27−30.33)	<0.001	18.31 (12.49−26.86)	<0.001
NLR						
≤2.139	148	287	1 (Ref)		1 (Ref)	
>2.139	58	595	5.29 (3.79−7.39)	<0.001	4.98 (3.54−7.01)	<0.001
PLR						
≤182.698	179	431	1 (Ref)		1 (Ref)	
>182.698	28	450	6.68 (4.39−10.16)	<0.001	6.46 (4.22−9.88)	<0.001
LMR						
>3.619	171	409	1 (Ref)		1 (Ref)	
≤3.619	35	470	5.61 (3.81−8.27)	<0.001	5.30 (3.58−7.85)	<0.001
**Early stage**						
CA125						
≤35	141	71	1 (Ref)		1 (Ref)	
>35	58	211	7.23 (4.81−10.86)	<0.001	6.66 (4.41−10.05)	<0.001
NLR						
≤2.139	148	158	1 (Ref)		1 (Ref)	
>2.139	58	140	2.26 (1.55−3.30)	<0.001	2.16 (1.48−3.17)	<0.001
PLR						
≤182.698	179	220	1 (Ref)		1 (Ref)	
>182.698	28	78	2.27 (1.41−3.64)	0.001	2.28 (1.41−3.70)	0.001
LMR						
>3.619	171	200	1 (Ref)		1 (Ref)	
≤3.619	35	96	2.35 (1.51−3.63)	<0.001	2.36 (1.51−3.69)	<0.001
**Advantaged stage**						
CA125						
≤35	141	16	1 (Ref)		1 (Ref)	
>35	58	524	79.62 (44.41−142.75)	<0.001	71.89 (39.62−130.45)	<0.001
NLR						
≤2.139	148	123	1 (Ref)		1 (Ref)	
>2.139	58	440	9.13 (6.35−13.13)	<0.001	9.26 (6.31−13.58)	<0.001
PLR						
≤182.698	179	200	1 (Ref)		1 (Ref)	
>182.698	28	362	11.57 (7.50−17.86)	<0.001	11.86 (7.57−18.56)	<0.001
LMR						
>3.619	171	197	1 (Ref)		1 (Ref)	
≤3.619	35	366	9.08 (6.07−13.58)	<0.001	8.82 (5.63−12.90)	<0.001

■ Benign ■ Malignant

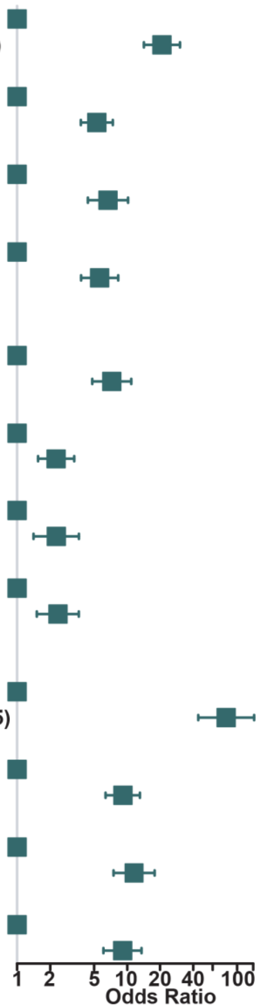

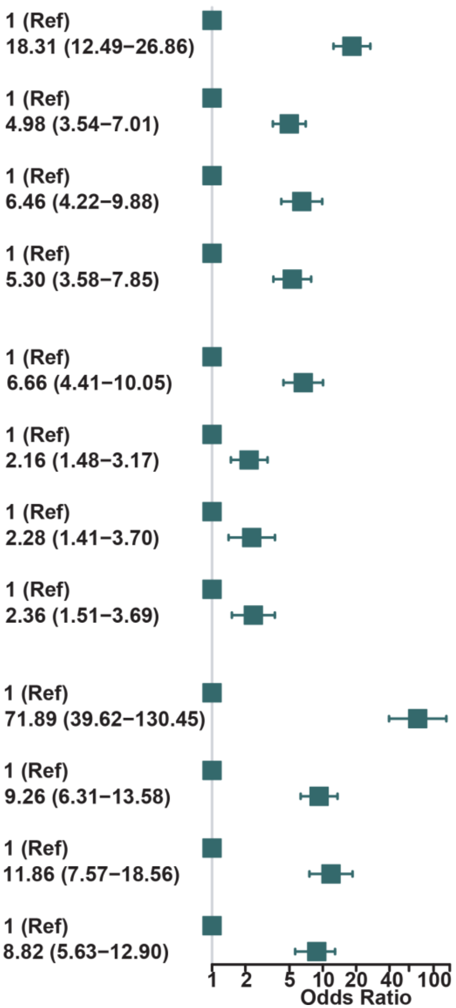

Figure S6.

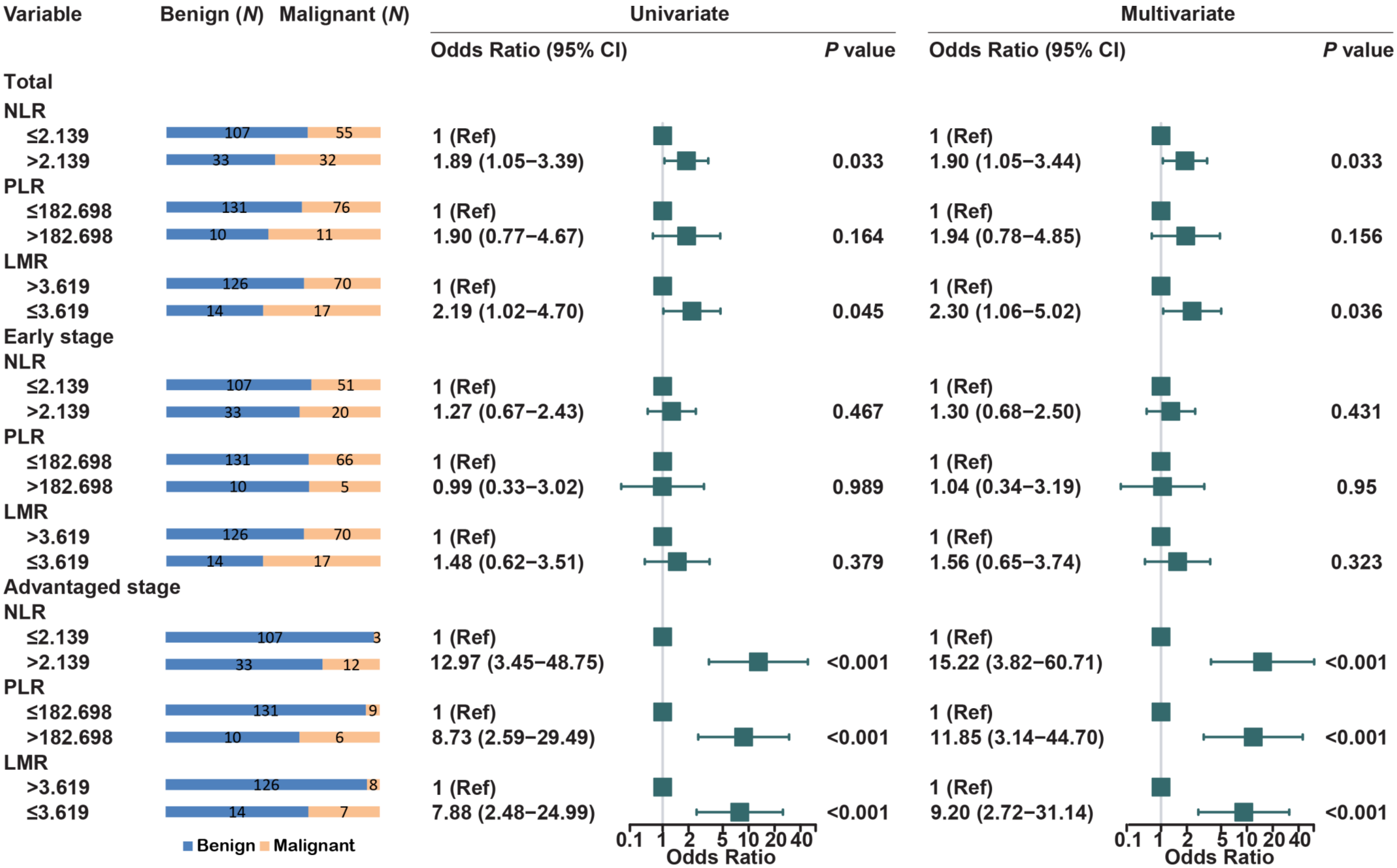
Variable
Benign (N)
Malignant (N)
Univariate
Multivariate
Odds Ratio (95% CI)
P value
Odds Ratio (95% CI)
P value
Total
NLR
≤2.139 107 55 1 (Ref) 1 (Ref)
>2.139 33 32 1.89 (1.05−3.39) 0.033 1.90 (1.05−3.44) 0.033
PLR
≤182.698 131 76 1 (Ref) 1 (Ref)
>182.698 10 11 1.90 (0.77−4.67) 0.164 1.94 (0.78−4.85) 0.156
LMR
>3.619 126 70 1 (Ref) 1 (Ref)
≤3.619 14 17 2.19 (1.02−4.70) 0.045 2.30 (1.06−5.02) 0.036
Early stage
NLR
≤2.139 107 51 1 (Ref) 1 (Ref)
>2.139 33 20 1.27 (0.67−2.43) 0.467 1.30 (0.68−2.50) 0.431
PLR
≤182.698 131 66 1 (Ref) 1 (Ref)
>182.698 10 5 0.99 (0.33−3.02) 0.989 1.04 (0.34−3.19) 0.95
LMR
>3.619 126 70 1 (Ref) 1 (Ref)
≤3.619 14 17 1.48 (0.62−3.51) 0.379 1.56 (0.65−3.74) 0.323
Advantaged stage
NLR
≤2.139 107 3 1 (Ref) 1 (Ref)
>2.139 33 12 12.97 (3.45−48.75) <0.001 15.22 (3.82−60.71) <0.001
PLR
≤182.698 131 9 1 (Ref) 1 (Ref)
>182.698 10 6 8.73 (2.59−29.49) <0.001 11.85 (3.14−44.70) <0.001
LMR
>3.619 126 8 1 (Ref) 1 (Ref)
≤3.619 14 7 7.88 (2.48−24.99) <0.001 9.20 (2.72−31.14) <0.001
Benign
Malignant
0.1 1 2 5 10 20 40
Odds Ratio
0.1 1 2 5 10 20 40
Odds Ratio

Figure S7.

Variable	Benign (*N*)	Malignant (*N*)	Univariate Odds Ratio (95% CI)	Univariate *P* value	Multivariate Odds Ratio (95% CI)	Multivariate *P* value
**Total**						
NLR						
≤2.139	34	218	1 (Ref)		1 (Ref)	
>2.139	24	531	3.45 (2.00−5.96)	<0.001	3.43 (1.98−5.94)	<0.001
PLR						
≤182.698	40	332	1 (Ref)		1 (Ref)	
>182.698	18	417	2.79 (1.57−4.96)	<0.001	2.85 (1.60−5.07)	<0.001
LMR						
>3.619	39	319	1 (Ref)		1 (Ref)	
≤3.619	19	427	2.75 (1.56−4.85)	<0.001	2.73 (1.54−4.82)	0.001
**Early stage**						
NLR						
≤2.139	34	99	1 (Ref)		1 (Ref)	
>2.139	24	112	1.60 (0.89−2.89)	0.116	1.55 (0.86−2.80)	0.148
PLR						
≤182.698	40	143	1 (Ref)		1 (Ref)	
>182.698	18	68	1.06 (0.57−1.98)	0.863	1.09 (0.58−2.06)	0.779
LMR						
>3.619	39	129	1 (Ref)		1 (Ref)	
≤3.619	19	80	1.27 (0.69−2.36)	0.442	1.31 (0.71−2.44)	0.389
**Advantaged stage**						
NLR						
≤2.139	34	115	1 (Ref)		1 (Ref)	
>2.139	24	405	4.99 (2.84−8.75)	0.001	5.21 (2.94−9.23)	<0.001
PLR						
≤182.698	40	180	1 (Ref)		1 (Ref)	
>182.698	18	340	4.20 (2.34−7.53)	<0.001	4.30 (2.38−7.77)	<0.001
LMR						
>3.619	39	181	1 (Ref)		1 (Ref)	
≤3.619	19	339	3.84 (2.16−6.85)	<0.001	3.75 (2.09−6.71)	<0.001

■ Benign ■ Malignant

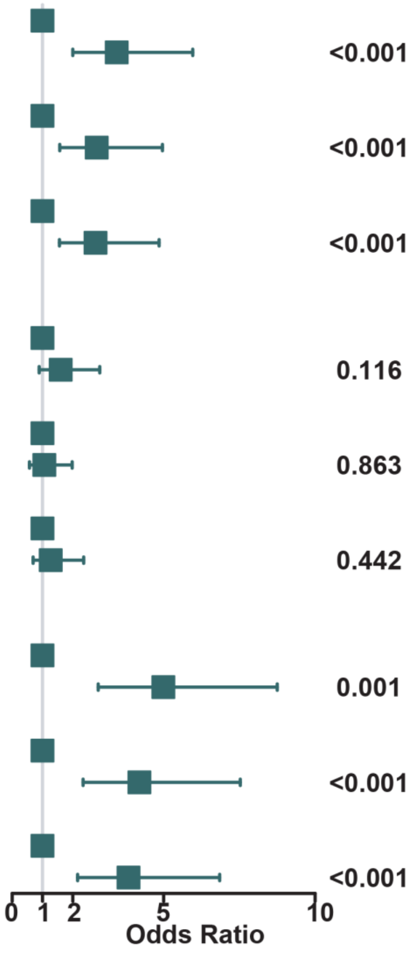

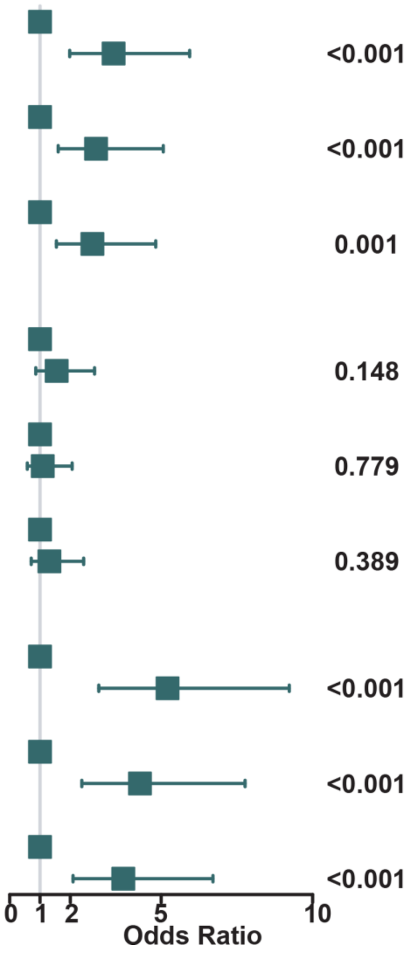

Supplement: Supplementary file 1 — Supplementary figures and tables. [file jcav12p2687s1.pdf]
